# Gadoxetic acid–enhanced MRI with a focus on LI-RADS v2018 imaging features predicts the prognosis after radiofrequency ablation in small hepatocellular carcinoma

**DOI:** 10.3389/fonc.2023.975216

**Published:** 2023-02-02

**Authors:** Ruizhi Wang, Hengtian Xu, Wufei Chen, Liang Jin, Zhuangxuan Ma, Lei Wen, Hongwei Wang, Kun Cao, Xia Du, Ming Li

**Affiliations:** ^1^ Department of Radiology, Huadong Hospital, Fudan University, Shanghai, China; ^2^ Department of Radiology, The Affiliated Hospital of Guizhou Medical University, Guiyang, China; ^3^ Department of General Surgery, Huadong Hospital, Fudan University, Shanghai, China; ^4^ Department of Hepatobiliary Surgery, The Affiliated Hospital of Guizhou Medical University, Guiyang, China

**Keywords:** small hepatocellular carcinoma, radiofrequency ablation, gadoxetic acid-enhanced MRI, nomogram, prognosis

## Abstract

**Introduction:**

Gadoxetic acid–enhanced magnetic resonance imaging (MRI) contributes to evaluating the prognosis of small hepatocellular carcinoma (sHCC) following treatment. We have investigated the potential role of gadoxetic acid–enhanced MRI based on LI-RADS (Liver Imaging Reporting and Data System) v2018 imaging features in the prognosis prediction of patients with sHCC treated with radiofrequency ablation (RFA) as the first-line treatment and formulated a predictive nomogram.

**Methods:**

A total of 204 patients with sHCC who all received RFA as the first-line therapy were enrolled. All patients had undergone gadoxetic acid–enhanced MRI examinations before RFA. Uni- and multivariable analyses for RFS were assessing using a Cox proportional hazards model. A novel nomogram was further constructed for predicting RFS. The clinical capacity of the model was validated according to calibration curves, the concordance index (C-index), and decision curve analyses.

**Results:**

Alpha fetoprotein (AFP) > 100 ng/ml (HR, 2.006; 95% CI, 1.111–3.621; P = 0.021), rim arterial phase hyperenhancement (APHE) (HR, 2.751; 95% CI, 1.511–5.011; P = 0.001), and targetoid restriction on diffusion-weighted imaging (DWI) (HR, 3.289; 95% CI, 1.832–5.906; P < 0.001) were considered as the independent risk features for recurrence in patients with sHCC treated with RFA. The calibration curves and C-indexes (C-index values of 0.758 and 0.807) showed the superior predictive performance of the integrated nomogram in both the training and validation groups.

**Discussion:**

The gadoxetic acid–enhanced MRI features based on LI-RADS v2018, including rim APHE, targetoid restriction on DWI, and the AFP level, are the independent risk factors of recurrence in patients with sHCC treated with RFA as the first-line therapy. The predictive clinical-radiological nomogram model was constructed for clinicians to develop individualized treatment and surveillance strategies.

## Introduction

Hepatocellular carcinoma (HCC), the sixth leading cause of cancer-related deaths in the world, is also one of the most common malignant tumors (1). Nowadays, not only surgical resection but also more treatment strategies, such as transarterial chemoembolization (TACE), ablation therapy, cryotherapy, and immune checkpoint inhibitors therapy, can be applied to HCC (2–4). Among them, radiofrequency ablation (RFA) has become one of the most commonly used first-line therapies for small HCC (sHCC) (5–7). There are increasingly more studies showing the safety and effectiveness of RFA for small or early-stage HCC (8, 9). However, recurrence and metastasis have still been occurred in the patients with sHCC treated with RFA. Several previous literature studies have reported that the 1-year recurrence rate of patients with sHCC treated with ablation was approximately from 10% to 30% (10, 11). However, another study recently reported by Joo Hyun Oh showed that recurrence-free survival (RFS) and overall survival (OS) at 5-year are only 72.3% and 22.0% for patients with sHCC treated with RFA (12). Thus, preoperative prognosis evaluation for patients treated with RFA as first-line therapy is urgently needed to formulate further individual treatment strategies.

Preoperative imaging examinations, especially magnetic resonance imaging (MRI), play an important role in the prognosis prediction of patients with HCC treated with surgical resection or interventional therapy (12–14). More interestingly, a kind of hepatocyte-specific contrast agents, gadoxetic acid (Gd-EOB-DTPA or Primovist), has been emerged and widely applied, showing an extremely promising value in the diagnosis and prognosis assessment of liver tumors (15, 16). In addition, compared with traditional contrast agent, gadoxetic acid–enhanced MRI is beneficial for the accurate and early diagnosis of sHCC nodules during surveillance as previously demonstrated (17, 18). Emerging pieces of studies suggested that several imaging features and ready availability from the gadoxetic acid–enhanced MRI make it an excellent candidate parameter for prognosis prediction of patients with HCC treated with surgical resection or interventional therapy (19). Unfortunately, the standard use and repeatability of various imaging features from gadoxetic acid–enhanced MRI in the prognosis assessment of HCC are still lacking, which is one of the reasons why these so-called imaging features cannot be formally recognized and applied in clinical practice. The LI-RADS (Liver Imaging Reporting and Data System) was formulated, aiming to promote a standard imaging-based diagnosis of HCC. Several emerging studies have reported that the LI-RADS system is associated with the prognosis of patients with primary HCC treated with surgical resection, independent of the pathologic diagnosis (20, 21). The establishment of the prognostic model based on standard imaging features is of great significance for radiologists/clinicians to routinely use and popularize in clinic. However, to our knowledge, few studies have focused on the gadoxetic acid–enhanced MRI based on LI-RADS v2018 (a latest version) imaging features that predict the prognosis after RFA as a first-line therapy in sHCC.

Hence, the purpose of our study was to mainly evaluate the potential role of LI-RADS v2018 (a latest version) imaging features from gadoxetic acid–enhanced MRI in the prediction of prognosis in patients with sHCC treated with RFA as first-line treatment. Furthermore, a nomogram model integrating standard imaging parameters and clinical parameters was developed for improving the practicability and repeatability of imaging in prognosis evaluation of patients with sHCC treated with ablation.

## Methods

### Patient selection

**Figure 1:**
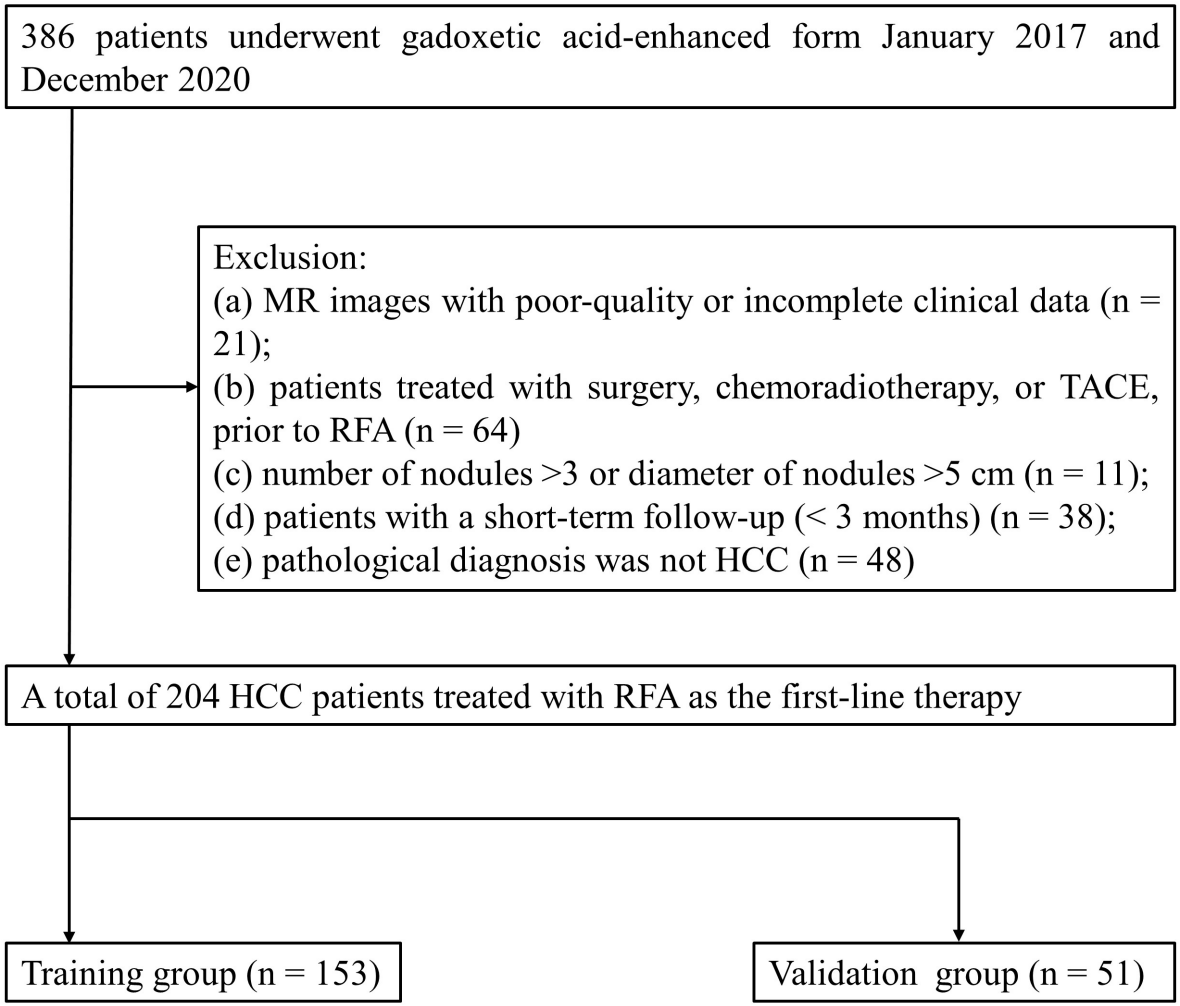
The flowchart of selection of patients with sHCC.

The retrospective study was approved by the institutional review board of our institution. The corresponding requirement for informed consent was waived. Between January 2017 and December 2020, a total of 386 patients treated with RFA were retrospectively analyzed. The inclusion criteria are as follows: (1) patients who underwent preoperative gadoxetic acid–enhanced MRI were diagnosed with HCC, and the MRI examinations were conducted within 2 weeks prior to RFA; (2) a single HCC ≤ 5 cm in diameter or up to three HCCs that were each ≤ 3 cm in diameter without portal vein thrombosis or extrahepatic metastases; (3) the coagulation function met the operation requirements; and (4) patients who did not undergo other treatment prior to surgery and have no history of extrahepatic cancer. The exclusion criteria are as follows: (a) MR images with poor-quality or incomplete clinical data; (b) patients treated with surgery, chemoradiotherapy, or TACE prior to RFA; (c) number of nodules > 3 or diameter of nodules > 5 cm; (d) patients with a short-term follow-up (<3 months); and (e) pathological diagnosis was not HCC. The first-line therapy was defined as no prior therapy for patients with sHCCs at the time of the first diagnosis. Finally, 204 patients with sHCCs treated with RFA as first-line therapy were enrolled in this study. The flow chart is shown in detail in **Figure 1**.

### MRI protocols

All patients in this study were examined in a 3.0-T MRI scanner (Canon Vantege Titan) equipped with a phased-array body coil before RFA. MRI sequences contained conventional in- and opposed-phase sequences, T1WI, fat-suppressed T2WI, and diffusion-weighted imaging (DWI, b = 0, 1000). Dynamic contrast-enhanced MR imaging with a T1-weighted fat-suppressed sequence was obtained once before and four times after intravenous administration. Acquisitions were performed at 25, 90, and 180 s and 20 min after injection gadoxetic acid (Primovist; Bayer Healthcare, Berlin, Germany). During the contrast-enhanced MRI, all patients were treated with Primovist at a rate of 3 ml/s and at a dose of 0.0025 mmol/kg, immediately followed by a 25-ml saline flush using a power injector.

### RFA procedures

Percutaneous RFA was performed by under general anesthesia using an S-1500 radiofrequency therapeutic apparatus (MedSphere^®^ International, Shanghai, China). All interventional procedures were conducted by one of the two interventional radiologists who had 11 and 8 years of interventional operation experience, respectively. The negative patch was pasted to the skin of back, thigh, or waist because of sparse hair. The different models and specifications of ablation needles were chosen according to the size of tumor. Then, the needle tip was sent to distal edge of tumor, and the active umbrella-like structure of needle tip was put up to a certain degree based on the location of the tumor and needle. The initial power was set at 30 to 50 watts, and the ablation time was 6 to 10 min according to the chosen needles. The radiofrequency therapeutic apparatus would automatically stop when the targeted tissue impedance reached 500 Ω, and the tissue temperature reached 70°C to 100°C. If necessary, the position of the needle tip was adjusted, and the procedure was repeated to ensure the complete ablation of the tumor. The needle track was ablated to prevent track implantation and bleeding before the needle was pulled out. The RFA was finished when the hyperechoic ablation surrounding was large enough to cover the entire tumor and the ablative margin (at least 5 mm of normal liver parenchyma surrounding the tumor).

### Follow-up and outcome analysis

After RFA, the ultrasound (US), contrast-enhanced CT/MRI, and serum alpha fetoprotein (AFP) examination were performed every 3 months for the first year and then every 4–6 months thereafter. The outcome in this study was assessed by RFS. According to previous literature, the RFS was considered as the interval between the initial date of interventional therapy and the date of the first tumor recurrence or last follow-up visit before 1 October 2021. In our study, the tumor recurrence was defined as local recurrence (LR) and intrahepatic distant recurrence (IDR), and extrahepatic metastasis. Among them, LR was considered as the appearance of new tumor nodules at the surrounding of ablation zone, and IDR was considered as the appearance of new tumor nodules not around the ablation area.

### Clinical-radiological characteristics analysis

In this study, various clinical data—including age, sex, cause of underlying liver disease (chronic hepatitis B/chronic hepatitis C/alcoholic liver disease), Child–Pugh score (A/B), number of tumors, and tumor size—were recorded. In addition, a series of laboratory findings—including albumin, total bilirubin, prothrombin time–international normalized ratio (PT-INR), AFP, and protein induced by vitamin K absence or antagonist-II (PIVKA-II)—were simultaneously analyzed.

As for radiological findings, two radiologists (with 12 and 19 years of experience in abdominal imaging, respectively) evaluated the MRI images on a picture archiving communication system based on the LI-RADS v2018, which represented a standard description of terminology and criteria for interpretation of liver observations. Another observer (with more than 30 years of experience in abdominal diagnostic) was invited for an opinion when there was inconsistency, and a majority decision was finally obtained and served for further study.

According to LI-RADS v2108 diagnostic algorithm, arterial phase hyperenhancement (APHE) (rim/non-rim), washout (not peripheral/peripheral), enhancing capsule, delayed central enhancement, targetoid restriction on DWI and targetoid hepatobiliary phase (HBP) appearance, ancillary imaging features (including corona enhancement, fat sparing in solid mass, restricted diffusion, mild-moderate T2 hyperintensity, iron sparing in solid mass, HBP hypointensity, nodule-in-nodule architecture, mosaic architecture, fat in mass, more than adjacent liver, and blood products in mass), and LI-RADS categorization (LR-3/4/5/M) were analyzed and recorded. Moreover, tumor size (the maximum diameter of the tumor); tumor margins (well-/ill-defined); signal intensity (SI) on T1-weighted, T2-weighted; and arterial, portal venous, and delayed phase images were also analyzed. The SI in this study was recognized as hyperintense, hypointense, or isointense compared with the adjacent hepatic parenchyma.

### Construction and validation of the nomogram

Gadoxetic acid–enhanced MRI features based on LI-RADS v2018 diagnostic algorithm, served as predictive risk factors, were build the preliminary predictive model of the prognostic of sHCC after RFA. In addition, the utility of the preliminary MRI-based nomogram was verified by the calibration curve and concordance index (C-index). Moreover, the decision curve analysis (DCA) was conducted to determine the clinical utility of the nomogram *via* calculating the net benefits at various threshold probabilities.

### Statistical analysis

Statistical analysis was conducted using SPSS 26.0 (IBM, Armonk, NY) and R project version 4.1.2 (http://www.r-project.org/). The categorical variables were showed as numbers (percentages). Uni- and multivariable analyses for RFS were assessed using the Cox proportional hazards model, and multicollinearity test was simultaneously conducted. The survival curve was conducted by Kaplan–Meier analyses *via* the log-rank test. Moreover, the interobserver agreement on LI-RADS v2018 imaging features in our study was valuated using the Cohen κ coefficient. The agreement was divided as five levels according to κ values as follows: poor (< 0.20), fair (0.21–0.40), moderate (0.41–0.60) and well (0.61–0.80), and excellent (0.81–1.00).

The predictive nomogram integrating LI-RADS v2018 imaging features and clinical parameters was formulated using in R project. The predictive performance of the constructed nomogram was evaluated by calibration curves and C-index. All tests were two-sided, and P-value < 0.05 was considered as statistically significance.

## Results

### Patients characteristics in both training and validation groups

This study enrolled 204 patients, which were divided into training (n = 153) and validation (n = 51) groups. There were no significant differences in age, sex, cause of underlying liver disease, Child–Pugh score, number of tumors, tumor sized, various laboratory data, and various conventional imaging features and LI-RADS v2018 imaging findings between the training and validation groups. All detailed data about patient characteristics in both groups are presented in **Table 1**. In addition, there was no significant difference between the training and validation groups. Among the overall cohort, the median follow-up was 20.3 months (range from 3.4 to 36.0 months). During the follow-up, the recurrence rate was 36.7% (75/204), observed in all patients, among which 10 patients (13.3%) showed LR, 57 patients (72.1%) showed IDR, and 6 (7.6%) had extrahepatic recurrence. Moreover, the 1- and 2-year RFS rates were 87.7% and 75.4%, respectively.

**Table 1:** The clinical, pathological, and radiological characteristics of the study population.

Variables	Training group (n = 153)	Validation group (n = 51)	P-value
Age (years)			0.290
<60	65 (42.5)	26 (51.0)	
≥60	88 (57.5)	25 (49.0)	
Sex (M/W)			0.686
Woman	76 (49.7)	27 (52.9)	
Man	77 (50.3)	24 (47.1)	
Cause of underlying liver disease			0.105
Chronic hepatitis B	101 (66.0)	26 (51.0)	
Chronic hepatitis C	47 (30.7)	22 (43.1)	
Alcoholic liver disease	5 (3.3)	3 (5.9)	
Child–Pugh score			0.620
A	122 (79.7)	39 (76.5)	
B	31 (20.3)	12 (23.5)	
Number of tumors			0.656
1	110 (71.9)	35 (68.6)	
2–3	43 (28.1)	16 (31.4)	
Tumor size (cm)			0.417
≤3	108 (70.6)	39 (76.5)	
3–5	45 (29.4)	12 (23.5)	
Laboratory finding			
Albumin (g/dl)			0.169
Normal	76 (49.7)	31 (60.8)	
Abnormal	77 (50.3)	20 (39.2)	
Total bilirubin (mg/dl)			0.514
Normal	64 (41.8)	24 (47.1)	
Abnormal	89 (58.2)	27 (52.9)	
PT-INR			0.146
Normal	72 (47.1)	30 (58.8)	
Abnormal	81 (52.9)	21 (41.2)	
AFP (>100 ng/ml)			0.124
Normal	77 (50.3)	32 (62.7)	
Abnormal	76 (49.7)	19 (37.3)	
PIVKA-II (>40 mAU/ml)			0.808
Normal	75 (49.0)	26 (51.0)	
Abnormal	78 (51.0)	25 (49.0)	
Conventional imaging features			
Tumor margin			0.073
Well-defined	62 (40.5)	28 (54.9)	
Ill-defined	91 (59.5)	23 (45.1)	
Diffusion-weighted imaging			0.322
Hyperintense	113 (73.9)	34 (66.7)	
Iso-intense	40 (26.1)	17 (33.3)	
Hypointense	0 (0%)	0 (0%)	
Signal on arterial phase			0.354
Hypointense	47 (30.7)	11 (21.6)	
Isointense	50 (32.7)	18 (35.3)	
Hyperintense	56 (36.6)	22 (43.1)	
Signal on portal phase			0.590
Hypointense	52 (34.0)	20 (39.2)	
Isointense	51 (33.3)	16 (31.4)	
Hyperintense	50 (32.7)	15 (29.4)	
Signal on delayed phase			0.262
Hypointense	45 (29.4)	18 (35.3)	
Isointense	62 (40.5)	16 (31.4)	
Hyperintense	46 (30.1)	17 (33.3)	
LI-RADS v2018 imaging findings			
APHE			0.727
Non-rim APHE	104 (68.0)	36 (70.6)	
Rim APHE	49 (32.0)	15 (29.4)	
Washout			0.851
Not peripheral Washout	115 (75.2)	39 (76.5)	
Peripheral Washout	38 (24.8)	12 (23.5)	
Enhancing capsule			0.195
Presence	77 (50.3)	31 (60.8)	
Absence	76 (49.7)	20 (39.2)	
Delayed central enhancement			1.000
Presence	114 (74.5)	38 (74.5)	
Absence	39 (25.5)	13 (25.5)	
Targetoid restriction on DWI			0.667
Presence	104 (68.0)	33 (64.7)	
Absence	49 (32.0)	18 (35.3)	
Targetoid HBP appearance			0.703
Presence	118 (77.1)	38 (74.5)	
Absence	35 (22.9)	13 (25.5)	
Corona enhancement			1.000
Presence	120 (78.4)	40 (78.4)	
Absence	33 (21.6)	11 (21.6)	
Fat sparing in solid mass			0.920
Presence	122 (79.7)	41 (80.4)	
Absence	31 (20.3)	10 (19.6)	
Restrict diffusion			0.627
Presence	72 (47.1)	26 (51.0)	
Absence	81 (52.9)	25 (49.0)	
Mild-moderate T2 hyperintensity			0.284
Presence	122 (79.7)	37 (72.5)	
Absence	31 (20.3)	14 (27.5)	
Iron sparing in solid mass			0.766
Presence	120 (78.4)	41 (80.4)	
Absence	33 (21.6)	10 (19.6)	
Hepatobiliary phase hypointensity			0.513
Presence	113 (73.9)	40 (78.4)	
Absence	40 (26.1)	11 (21.6)	
Nodule-in-nodule architecture			0.092
Presence	125 (81.7)	36 (70.6)	
Absence	28 (18.3)	15 (29.4)	
Mosaic architecture			0.284
Presence	122 (79.7)	37 (72.5)	
Absence	31 (20.3)	14 (27.5)	
Fat in mass, more than adjacent liver			0.458
Presence	116 (75.8)	36 (70.6)	
Absence	37 (24.2)	15 (29.4)	
Blood products in mass			0.508
Presence	118 (77.1)	37 (72.5)	
Absence	35 (22.9)	14 (27.5)	
LI-RADS categorization			0.657
LR-3	29 (19.0)	8 (15.7)	
LR-4	34 (22.2)	12 (23.5)	
LR-5	63 (41.2)	21 (41.2)	
LR-M	27 (17.6)	10 (19.6)	

PT-INR, prothrombin time-international normalized ratio; alphafetoprotein, AFP, PIVKA-II, protein induced by vitamin K absence or antagonist-II; APHE, arterial phase hyperenhancement; DWI, diffusion weighted imaging; HBP, hepatobiliary phase; LI-RADS, Liver Imaging Reporting and Data System.

### Independent predictive radiological-clinical factors for RFS in patients with sHCC with RFA

**Figure 2:**
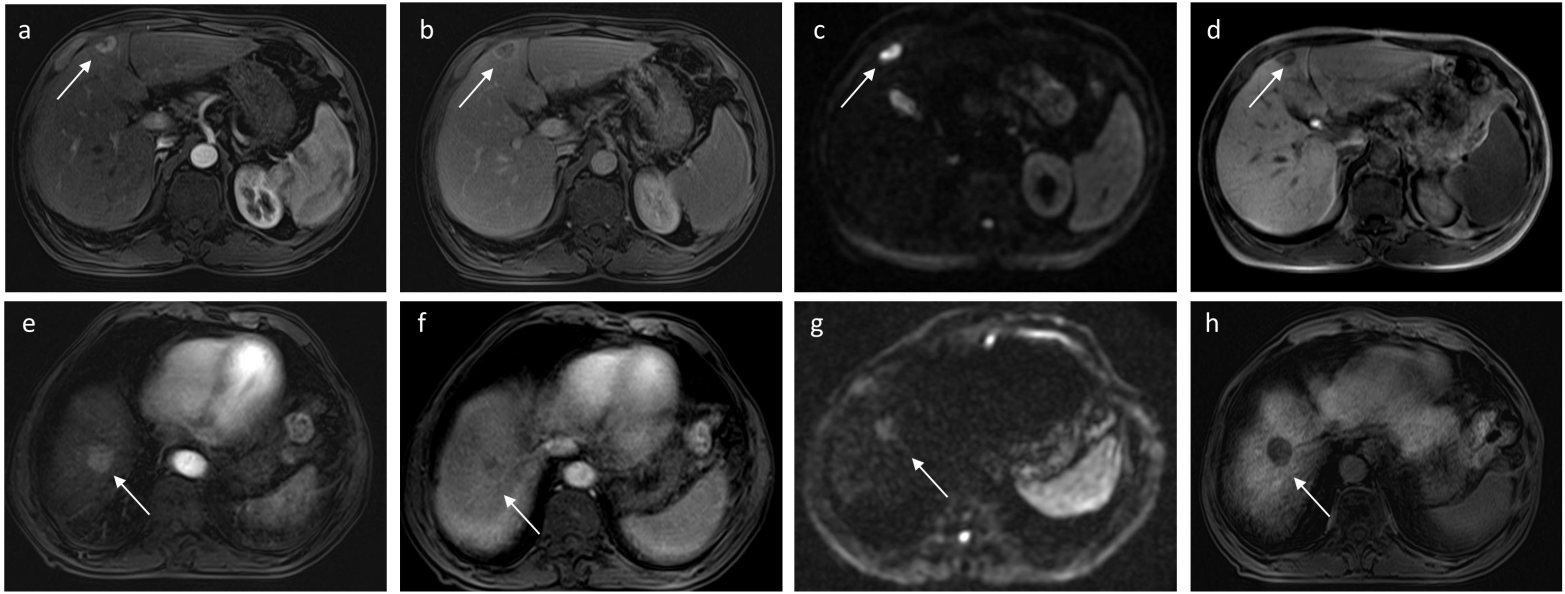
A 56-year-old man with sHCC was observed with early IDR after receiving RFA as the first-line therapy. Axial arterial **(A)** and delayed phases **(B)** show a well-defined tumor (white arrow) with rim APHE in hepatic segment IV. On the DWI **(C)**, the tumor exhibits targetoid restriction and the tumors showed targetoid HBP appearance on hepatobiliary phase **(D)**. Another 76-year-old man with HCC was observed with no recurrence after RFA during 13 months of the follow-up period. Axial arterial **(E)** and delayed phases **(F)** show a well-defined tumor (white arrow) with APHE in hepatic segment VIII. On the DWI **(G)**, the tumor exhibits appeared restriction, and the tumors exhibit completely hypointense on hepatobiliary phase **(H)**.

**Figure 3:**
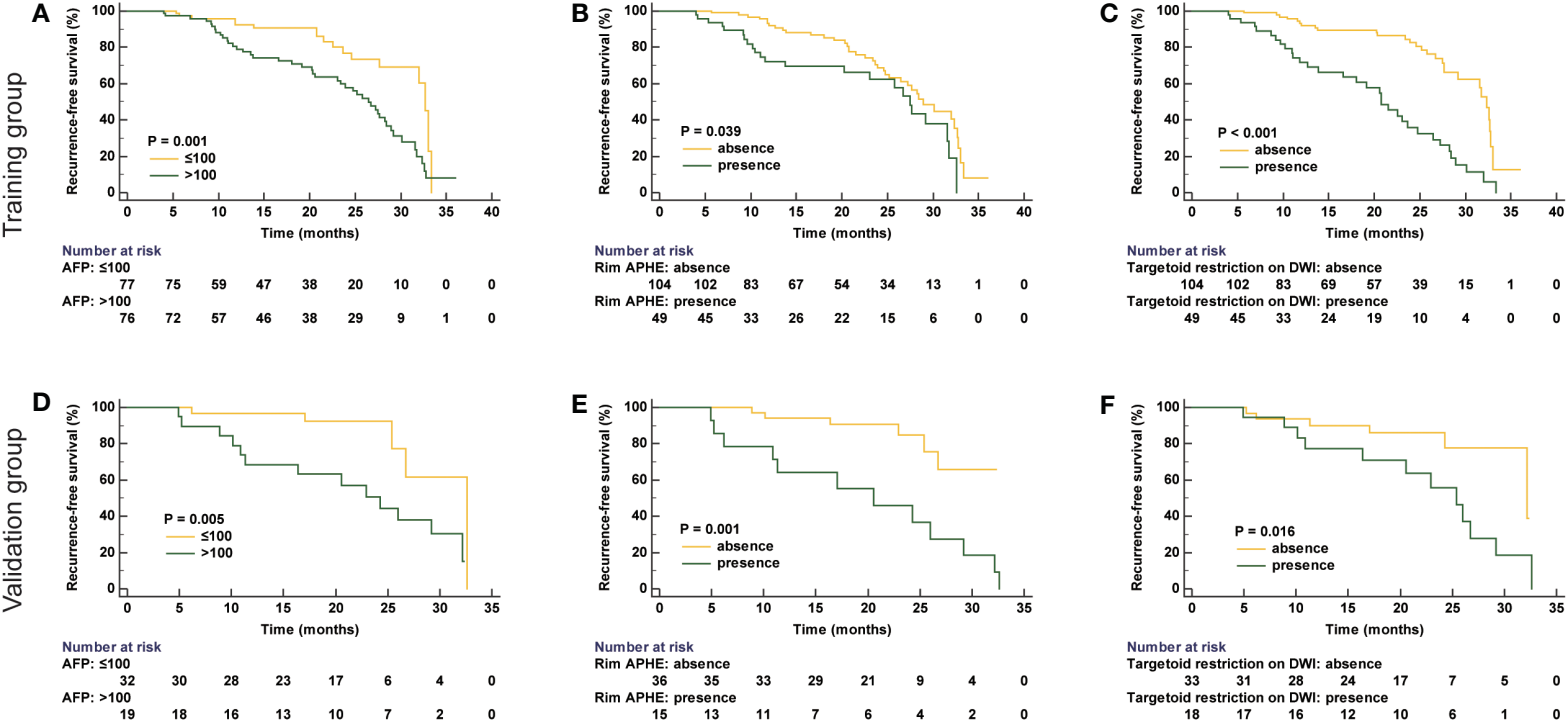
The RFS of each group in both training **(A–C)** and validation groups **(D–F)**.

In univariate logistic regression analysis, it is shown that tumor size, AFP level, rim APHE, targetoid restriction on DWI, and number of tumors were correlated with recurrence in the training group. Then, in the multivariate logistic regression analysis, AFP > 100 ng/ml (HR, 2.006; 95% CI, 1.111–3.621; P = 0.021), rim APHE (HR, 2.751; 95% CI, 1.511–5.011; P = 0.001), and targetoid restriction on DWI (HR, 3.289; 95% CI, 1.832–5.906; P < 0.001) were considered as the independent risk characteristics for recurrence in patients with sHCC treated with RFA as the first-line therapy (**Figures 2**, **3**). The detailed information is listed in **Table 2**. Moreover, to verify the complex collinearity among the variables, the multicollinearity test was performed, which showed that there was no multicollinearity among three variables. The variance inflation factors were 1.33, 1.21, and 1.17, respectively, which are all less than 5.

**Table 2:** Uni- and multivariate Cox analyses for risk factors for RFS in sHCC after RFA.

Parameters		Univariate analysis		Multivariate analysis	
		HR (95% CI)	P-value	HR (95% CI)	P-value
Age (years)	<60	Reference			
	≥60	1.395 (0.829–2.346)	0.210		
Sex	Female	Reference			
	Male	0.730 (0.439–1.214)	0.225		
Cause of underlying liver disease	Chronic hepatitis B	Reference			
	Chronic hepatitis C	0.880 (0.520–1.491)	0.635		
	Alcoholic liver disease	0.572 (0.134–2.437)	0.450		
Child–Pugh score	A	Reference			
	B	1.316 (0.706–2.453)	0.388		
Number of tumors	1	Reference			
	2–3	1.692 (1.004–2.852)	0.048*	0.916 (0.516–1.626)	0.765
Tumor size (cm)	≤3	Reference			
	3–5	1.872 (1.110–3.158)	0.019*	1.576 (0.927–2.680)	0.093
Laboratory finding					
Albumin (g/dl)	Normal	Reference			
	Abnormal	0.781 (0.468–1.303)	0.344		
Total bilirubin (mg/dl)	Normal	Reference			
	Abnormal	1.051 (0.623–1.772)	0.852		
PT-INR	Normal	Reference			
	Abnormal	1.189 (0.713–1.985)	0.507		
AFP (>100 ng/ml)	Normal	Reference			
	Abnormal	2.614 (1.473–4.640)	0.001*	2.006 (1.111–3.621)	0.021*
PIVKA-II (>40 mAU/mL)	Normal	Reference			
	Abnormal	1.158 (0.690–1.944)	0.579		
Conventional imaging features					
Tumor margin	Well-defined	Reference			
	Ill-defined	0.666 (0.398–1.114)	0.122		
Diffusion-weighted imaging	Hypointense	Reference			
	Hyperintense	0.872 (0.485-1.567)	0.646		
Signal on arterial phase	Hypointense	Reference			
	Isointense	0.771 (0.405–1.468)	0.429		
	Hyperintense	0.681 (0.373–1.245)	0.213		
Signal on portal phase	Hypointense	Reference			
	Isointense	1.088 (0.583–2.029)	0.791		
	Hyperintense	1.179 (0.623–2.231)	0.614		
Signal on delayed phase	Hypointense	Reference			
	Isointense	1.113 (0.591–2.097)	0.740		
	Hyperintense	1.164 (0.589–2.303)	0.662		
LI-RADS v2018 imaging findings					
APHE	Non-rim APHE	Reference			
	Rim APHE	2.047 (1.192–3.516)	0.009*	2.751 (1.511–5.011)	0.001*
Washout	Not peripheral washout	Reference			
	Peripheral washout	1.157 (0.653–2.053)	0.617		
Enhancing capsule	Presence	Reference			
	Absence	0.955 (0.571–1.596)	0.859		
Delayed central enhancement	Presence	Reference			
	Absence	1.381 (0.774–2.464)	0.275		
Targetoid restriction on DWI	Presence	Reference			
	Absence	2.833 (1.693–4.743)	< 0.001*	3.289 (1.832–5.906)	<0.001*
Targetoid HBP appearance	Presence	Reference			
	Absence	1.420 (0.806–2.504)	0.225		
Corona enhancement	Presence	Eeference			
	Absence	0.886 (0.478–1.642)	0.700		
Fat sparing in solid mass	Presence	Reference			
	Absence	1.588 (0.891–2.831)	0.117		
Restrict diffusion	Presence	Reference			
	Absence	1.043 (0.625–1.741)	0.872		
Mild-moderate T2 hyperintensity	Presence	Reference			
	Absence	0.702 (0.363–1.359)	0.294		
Iron sparing in solid mass	Presence	Reference			
	Absence	0.957 (0.509–1.798)	0.891		
Hepatobiliary phase hypointensity	Presence	Reference			
	Absence	0.890 (0.495–1.600)	0.697		
Nodule-in-nodule architecture	Presence	Reference			
	Absence	0.968 (0.475–1.975)	0.929		
Mosaic architecture	Presence	Reference			
	Absence	1.278 (0.697–2.346)	0.428		
Fat in mass, more than adjacent liver	Presence	Reference			
	Absence	1.071 (0.576–1.990)	0.829		
Blood products in mass	Presence	Reference			
	Absence	1.017 (0.561–1.845)	0.955		
LI-RADS categorization	LR-3	Reference			
	LR-4	1.062 (0.472–2.390)	0.885		
	LR-5	1.455 (0.709–2.986)	0.307		
	LR-M	0.990 (0.429–2.288)	0.982		

Bold represents statistically significant.

RFS, recurrence-free survival; sHCC, small hepatocellular carcinoma; RFA, radiofrequency ablation; PT-INR, prothrombin time-international normalized ratio; alphafetoprotein, AFP, PIVKA-II, protein induced by vitamin K absence or antagonist-II; APHE, arterial phase hyperenhancement; DWI, diffusion weighted imaging; HBP, hepatobiliary phase; LI-RADS, Liver Imaging Reporting and Data System.

In terms of the assessment of interobserver agreement of LI-RADS v2018 imaging features, the results showed that APHE (κ = 0.881), washout (κ = 0.910), enhancing “capsule” (κ = 0.811), delayed central enhancement (κ = 0.832), targetoid restriction on DWI (κ = 0.890), targetoid HBP appearance (κ = 0.865), mild-moderate T2 hyperintensity (κ = 0.882), HBP hypointensity (κ = 0.878), restricted diffusion (κ = 0.911), and blood products in mass (κ = 0.812) exhibited excellent interobserver agreement. Corona enhancement (κ = 0.871), fat sparing in solid mass (κ = 0.773), iron sparing in solid mass (κ = 0.767), nodule-in-nodule architecture (κ = 0.756), mosaic architecture (κ = 0.723), fat in mass, more than adjacent liver (κ = 0.798), and LI-RADS categorization (κ = 0.760) exhibited well interobserver agreement.

### Construction and validation of predictive nomogram for RFS

**Figure 4:**
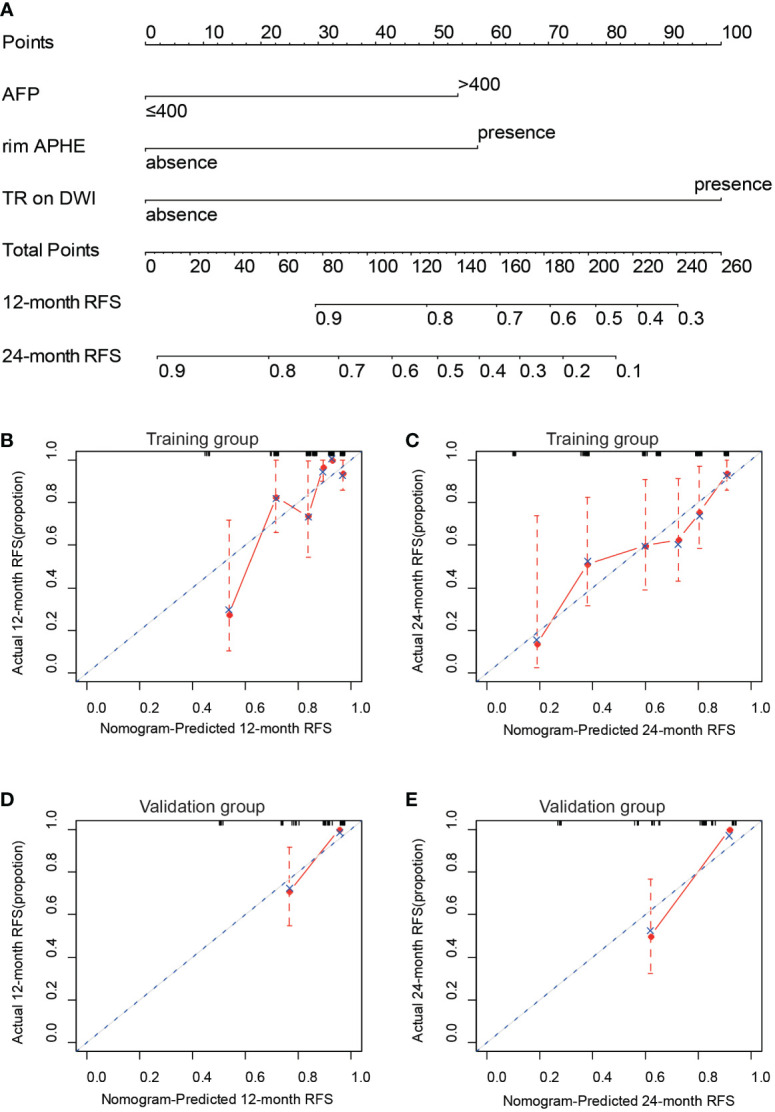
Construction and validation of nomogram for predicting RFS of patients with sHCC with RFA as the first-line therapy. **(A)** Integrated nomogram for predicting probability of 12- and 24-month after RFA of sHCC. The calibration curves for the nomogram-predicted 12-month RFS **(B)** and the nomogram-predicted 24-month RFS **(C)** in the training cohort and the nomogram-predicted 12-month RFS **(D)** and the nomogram-predicted 24-month RFS **(E)** in the validation cohort.

In this study, a predictive nomogram model including LI-RADS v2018 imaging findings and clinical features was established, which contains AFP > 100 ng/ml, rim APHE, and targetoid restriction on DWI for RFS after patients with sHCC treated with RFA as the first-line therapy (**Figure 4A**). The calibration curves of the developed nomogram for both the training and validation groups exhibited a good consistency between estimation and observation at 12 and 24 months after RFA (**Figures 4B–E**). As for C-index in the training group, the C-index for RFS prediction with the integrated nomogram (AFP + rim APHE + targetoid restriction on DWI) was 0.758 (95% CI, 0.679–0.837), which was higher than the C-index by other independent risk features. Similarly, in the validation group, the C-index for RFS prediction with the integrated nomogram (AFP + rim APHE + targetoid restriction on DWI) was 0.807 (95% CI, 0.712–0.904), showing the best prediction capacity (**Table 3**).

**Table 3:** Prediction performance of nomogram and independent risk factors in training and validation cohort.

Factors	Training group		Validation group	
	C-index	95% CI	C-index	95% CI
AFP	0.613	0.543–0.683	0.706	0.588–0.825
Rim APHE	0.585	0.511–0.659	0.709	0.584–0.833
Targetoid restriction on DWI	0.679	0.612–0.746	0.634	0.499–0.768
Nomogram (AFP + rim APHE)	0.653	0.569–0.736	0.794	0.703–0.885
Nomogram (AFP + targetoid restriction on DWI)	0.734	0.664–0.804	0.739	0.601–0.876
Nomogram (AFP + rim APHE + targetoid restriction on DWI)	0.758	0.679–0.837	0.807	0.712–0.904

C-index, concordance index; AFP, alphafetoprotein; APHE, arterial phase hyperenhancement; DWI, diffusion weighted imaging.

**Figure 5:**
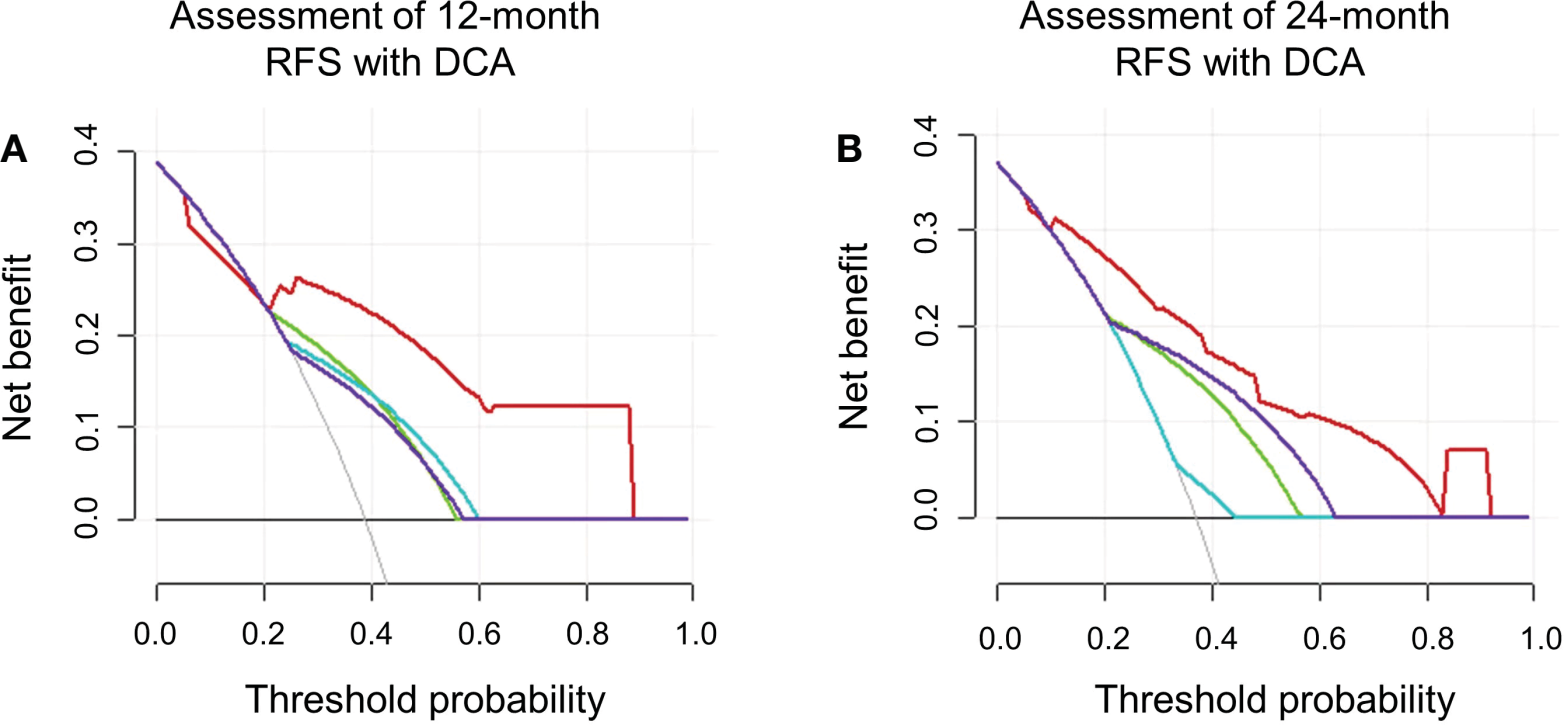
The decision curve analyses in terms of 12 **(A)** and 24 **(B)** months. RFS depicted integrated nomogram comprising the clinical feature (AFP) and LI-RADS v2018 imaging features on gadoxetic acid–enhanced MRI (rim APHE and targetoid restriction on DWI). Red line represents integrated nomogram (AFP + rim APHE + targetoid restriction on DWI); purple line represents targetoid restriction on DWI; green line represents AFP; blue line represents rim APHE.

On DCA, the developed nomogram showed a best net benefit with a wider range of threshold probability compared with the separate LI-RADS v2018 imaging findings and clinical features, indicating improved performance for predicting 12- and 24-month RFS (**Figure 5**).

## Discussion

In this study, we found that the clinical feature (AFP) and LI-RADS v2018 imaging features on gadoxetic acid–enhanced MRI (rim APHE and targetoid restriction on DWI) are the independent risk factors for recurrence of sHCC after RFA. Moreover, a novel integrated nomogram based on clinical parameters and MRI features was constructed to predict 12- and 24-month RFS of patients with sHCC after curative RFA as the first-line therapy.

As is known, LI-RADS category, a means of standard imaging-based diagnosis of HCC, has recently been applied in the classification or differentiation diagnosis of liver tumors (22). Intriguingly, in several previous studies, HCC with several imaging appearances from LI-RADS category, such as target-like imaging morphology is often closely associated with unfavorable biomarkers (9, 23). However, few studies have focused on the application of the LI-RADS category on the prognosis evaluation of sHCC treated with RFA as the first-line therapy. In our study, on the basis of LI-RADS v2018 category, we found that targetoid imaging features including rim APHE and targetoid restriction on gadoxetic acid–enhanced MRI were two of the valuable risk factors for recurrence of patients with sHCC following RFA. In addition, a combined nomogram model integrating LI-RADS imaging features in this study was constructed to enable clinicians to handily assess the individually recurrence risk of patients with sHCC, avoiding vague recurrence risk assessments or overly complicated recurrence risk calculations. According to the presented nomogram model, patients with a low recurrence risk are the optimal candidates of RFA as the first-line treatment. As to patients with HCC with a high recurrence risk, more therapies such as combined TACE and surgical resection may be the first choice. In addition, the postoperative follow-up needs to be earlier and more frequent for those patients.

In this study, rim APHE was one of the significant LI-RADS v2018 imaging features predictive of post-RFA recurrence of patients with sHCC. According to LI-RADS category, rim APHE was not a major feature of HCC, rather an imaging feature of cholangiocarcinoma or combined hepatocellular cholangiocarcinoma. In our study, 31.4% of patients in this cohort were showed rim APHE, which is slightly higher than 5%–13% reported in the previous literature (24). Although HCC presenting with rim APHE is relatively rare, HCC with rim APHE seems to be more invasive. A previous study has reported HCC with rim APHE expresses higher carbonic anhydrase IX and epithelial cell adhesion molecule levels, which are hypoxia- and stemness-related markers, respectively (25). Moreover, sHCCs presenting with rim APHE may be frequently associated with growth patterns and invasive pathophysiological features, such as microvascular invasion, abundant intratumoral fibrous stroma, tumor necrosis, and low microvascular density (25). As such, these aforementioned studies may explain our results that rim APHE was an independent recurrence predictor for patients with sHCC treated with RFA as the first-line therapy. In addition, similar results have been reported in some surgically resected HCC studies. For instance, Moon et al. have reported that HCCs categorized as LR-M with rim APHE often showed worst prognosis after surgical resection, meaning that rim APHE was helpful for assessing the postoperative prognosis of HCC after further stratification of LR-M on preoperative MRI (12). Moreover, in another recent study reported by Kang et al., rim APHE at gadoxetate-enhanced MRI was used to distinguish non-proliferative class HCC from proliferative class HCC, which was an independent factor for poor survival of HCC (26). In addition, this imaging feature can also be used to assess OS and incidence of extrahepatic metastasis in the proliferative class HCC.

Targetoid restriction on DWI was another LI-RADS v2018 imaging feature predictive of post-RFA recurrence of patients with sHCC. Similar to rim APHE, targetoid restriction on DWI, as one of the targetoid imaging appearances, was another typical imaging feature of non-HCC malignancies (27). Such imaging feature was showed as restricted diffusion in tumor periphery on DWI with less restricted diffusion in tumor center, which may reflect peripheral hypercellularity and central stromal fibrosis or ischemia (28). Moreover, the increased diffusivity of the loose fibrotic component with necrosis could be the main contributor for central darkness on DWI. Thus, the above histopathologic properties are highly correlated with targetoid restriction on DWI and consistent with our results showing that targetoid restriction was another independent risk factor for recurrence of sHCC after RFA. On the other hand, some studies have also shown that the targetoid restriction on DWI may be associated with some biologically invasive characteristics, such as CK-19. For example, Hu et al. have reported that MR features with targetoid appearances based on LI-RDAS v2017, such as targetoid appearance on DWI (P = 0.001), were more frequently observed in comparison with CK19-negative HCCs. These results may be the potential reason for worse post-RFA outcomes of patients with HCC presenting with targetoid restriction on DWI (25).

In addition to imaging features based on LI-RDAS v2018 in this study, clinical characteristics were incorporated into the predictive model for further improve the practicality and predictive efficiency of the developed nomogram in clinic. In our study, AFP (>100 ng/ml) was considered as a clinical independent risk factor for post-RFA recurrence of patients with sHCC. As is well known, AFP is one of the most commonly used markers of HCC in clinic (29, 30). Several present studies have increasingly showed that the AFP level was closely associated with the cellular differentiation, microvascular invasion, and tumor prognosis. For example, in a recent study, Hu et al. have reported that the AFP level was identified as one of the independent risk factors for early recurrence after ablation (10). However, in their study, the risk of postoperative recurrence was greatest when AFP was greater than 400 ng/ml. Similar results were seen in the assessment of outcomes after TACE of HCC (31). Song and coworkers have demonstrated that the AFP level (>13.2 ng/ml) was identified as clinical risk factor for recurrence after TACE of HCC (32). Note that AFP (≤100 ng/ml) was normal in more than 50% of patients in our cohort. With different cutoff values of AFP, the effectiveness of the AFP level in the prediction model will also change.

There were several limitations in this study. First, the present study is a retrospective and single-center study; therefore, reselection and verification biases were inevitable. Only patients with sHCC that fully met RFA as the first-line therapy were enrolled, and patients with poor-quality MR images or incomplete clinical data were excluded; thus, the results in our study may not represent the true spectrum of HCC after RFA. Moreover, in this study, sHCC (<5 cm) was included in the study. If we only conducted RFA treatment for patients with sHCC (<3 cm), then the clinical effect might be better (33). For patients with HCC of 3–5 cm, TACE + RFA may achieve better efficacy (34). Second, we only focused on the MRI features according to the LI-RDAS v2018 several MRI features reported in the previous literature such as peritumoral signal on different sequences that were not analyzed in our study. Further sophisticated investigations with more samples in the multicenters should be conducted. Third, only pre-RFA clinical-radiological features were analyzed, and the correlation analysis between detailed pathological characteristics and prognosis of HCC after RFA was lacking. Finally, for practicability in clinic and easy popularity, the predictive models were only integrating the conventional and standard MRI features. In addition, genomic directed stratifications in clinical trial design are needed to be considered in future study.

In conclusion, the predictive model based on LI-RADS v2018 MRI features and clinical factors could be used to assess the prognosis of patients before RFA as the first-line treatment, which contributed to screening out the high probability of recurrence in patients with sHCC treated with RFA. Moreover, such an integrated nomogram may be used as a convenient method for facilitating clinicians to make precise and personalized management decisions.

## Data availability statement

The original contributions presented in the study are included in the article/supplementary material. Further inquiries can be directed to the corresponding authors.

## Author contributions

RW, HX, and ML designed this study. RW, HX, WC, LJ, and ZM collected the patients’ data. LW, HW, and KC conducted the statistical analysis of this study. RW and HX wrote the manuscript. ML revised and supervised the manuscript. All authors contributed to the article and agreed to the submitted version.
